# Correction: Effect of cutting depth during sugarcane (*Saccharum* spp. hybrid) harvest on root characteristics and yield

**DOI:** 10.1371/journal.pone.0248527

**Published:** 2021-03-08

**Authors:** Shao-lin Yang, Yue-bin Zhang, Jun Deng, Ru-dan Li, Xian Fan, Jing-mei Dao, Yi-ji Quan, Syed Asad Hussain Bukhari

In Fig 6, the -10cm legend is missing. Please see the correct Fig 6 here.

**Fig 6:**
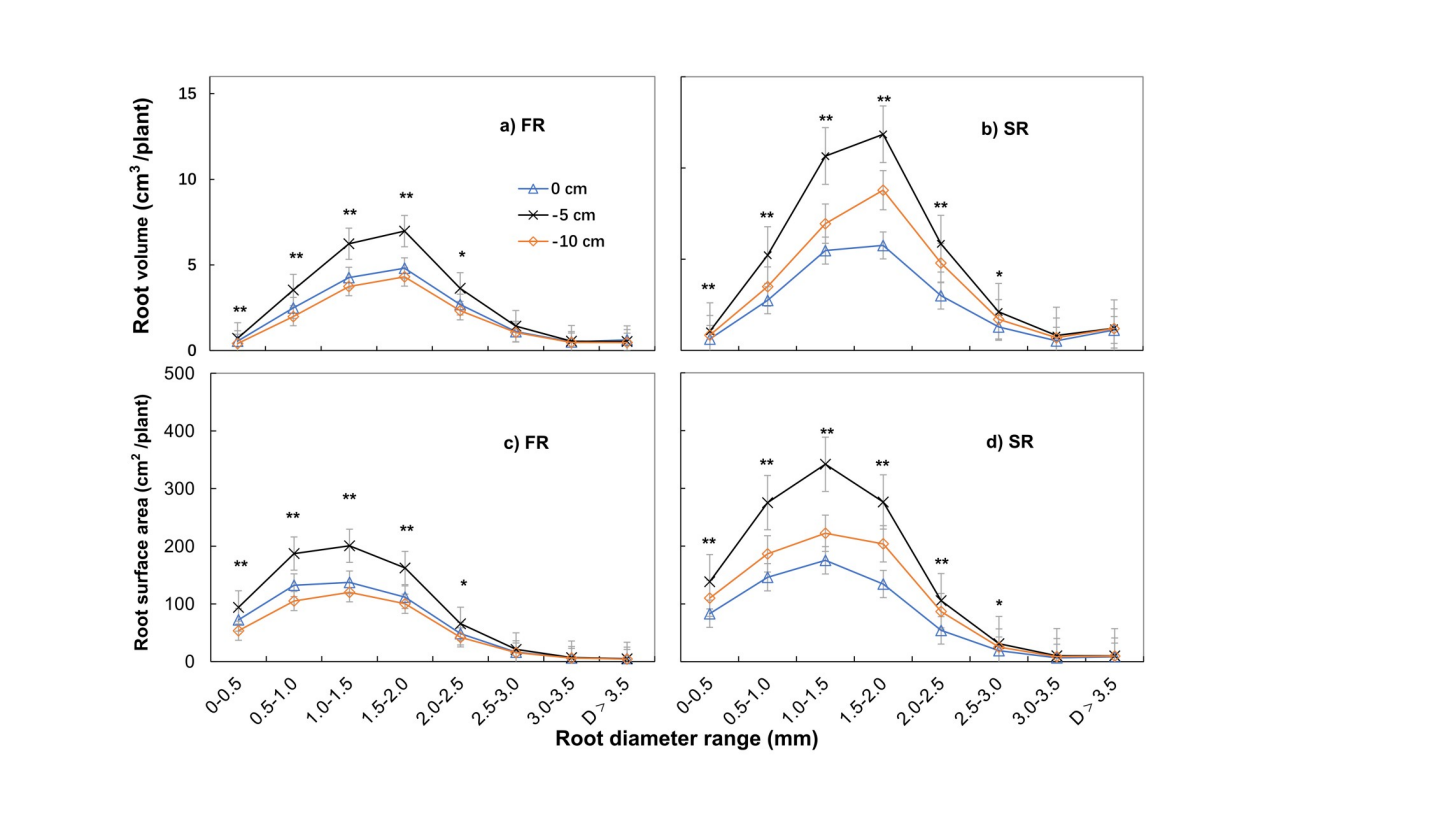
Effect of cutting depth on root volume and root surface area according to root diameter (D) in the first (FR) and second ratoon crop (SR). Root volume was highest in roots with a diameter of 1.5–2.0 mm in both FR and SR, while the root surface area was highest at a diameter of 1.0–1.5 mm plant^-1^: the amount in each cluster/ millable canes in the cluster. * P < 0.05, ** P < 0.01. Error bars, SD.

In the Root characteristics under each cutting depth according to root diameter subsection of the Results, there is an error in the second sentence of the third paragraph. The correct sentence is: Accordingly, the root fresh weight ranged from 167–287 g m^-2^, root volume from 79–164 cm^3^ m^-2^, root surface area from 3628–7329 cm^2^ m^-2^, root length from 13,430–26,349 cm m^-2^, root tip number from 36,538–68,473 m^-2^, root forks number from 114,364–215,035 m^-2^, and root crossings from 9,059–16,704 m^-2^.
