# Supplementary figures and images for: Effect of cutting depth during sugarcane (Saccharum spp. hybrid) harvest on root characteristics and yield
Source: PLoS One. 2021 Jan 22;16(1):e0238085. doi: 10.1371/journal.pone.0238085 (PMC7822348; doi:10.1371/journal.pone.0238085)

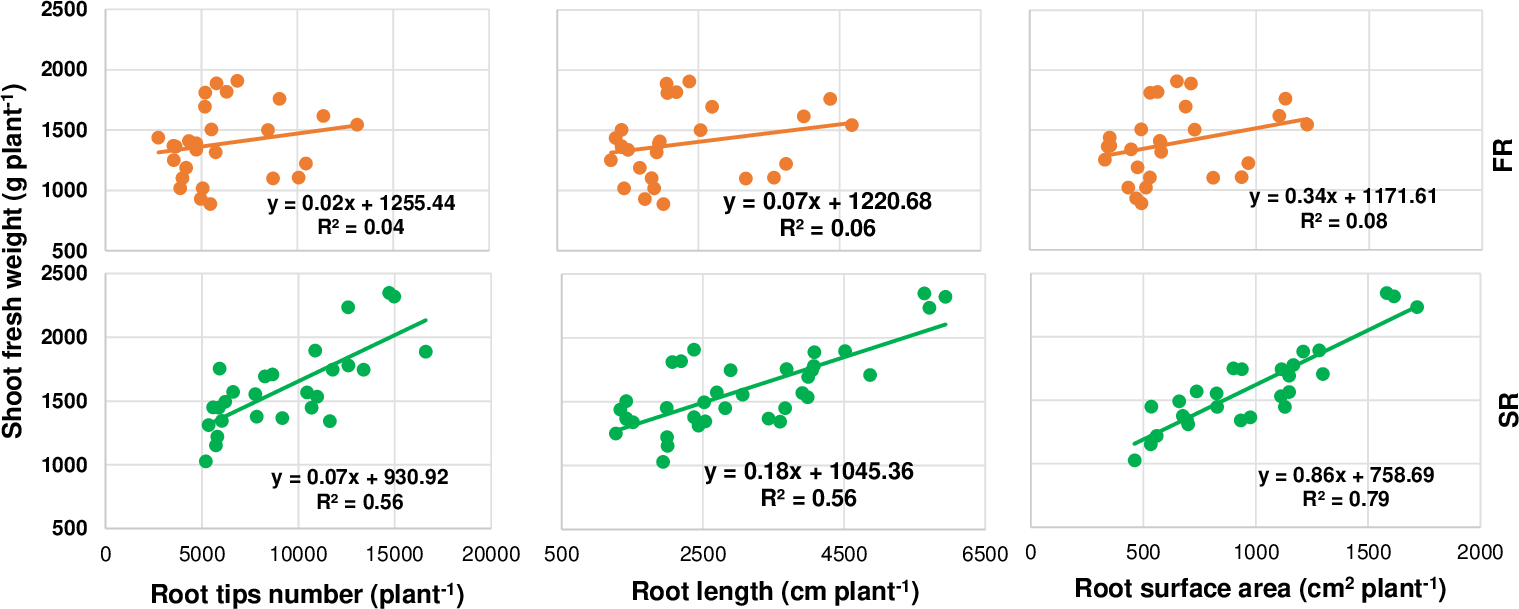

Supplement: S1 Fig — Plant-1: the amount per each cluster / millable canes in the cluster. (TIF) [file pone.0238085.s001.tif]
