# Supplementary material for: Effect of cutting depth during sugarcane (Saccharum spp. hybrid) harvest on root characteristics and yield
Source: PLoS One. 2021 Jan 22;16(1):e0238085. doi: 10.1371/journal.pone.0238085 (PMC7822348; doi:10.1371/journal.pone.0238085)
Supplement: S1 Table — RFW: root fresh weight; RDW: root dry weight; SFW: Shoot fresh weight; SDW: Shoot dry weight; RL: root length; RSA: root surface area; RAD: root average diameter; RLPV: root length per volume; RV: root volume. First ratoon crop (FR) n = 53, second ratoon crop (SR) n = 43. ** P<0.01, * P<0.05 (2-tailed). (DOCX) [file pone.0238085.s002.docx]

**Table S1.** Correlation between root morphology and shoots biomass. RFW: root fresh weight; RDW: root dry weight; SFW: Shoot fresh weight; SDW: Shoot dry weight; RL: root length; RSA: root surface area; RAD: root average diameter; RLPV: root length per volume; RV: root volume. First ratoon crop (FR) n=53, second ratoon crop (SR) n=43. ** P＜0.01, * P＜0.05 (2-tailed).

|  | **Ratoon** | **RFW(g)** | **RDW(g)** | **SFW(g)** | **SDW(g)** | **RL (cm)** | **RSA (cm^2^)** | **RAD (mm)** | **RLPV**  **(cm m^-3^)** | **RV (cm^3^)** | **Tips** | **Forks** | **Crossings** |
| --- | --- | --- | --- | --- | --- | --- | --- | --- | --- | --- | --- | --- | --- |
| **RFW(g)** | **FR** | **1** | **0.967^**^** | **0.859^**^** | **0.841^**^** | **0.836^**^** | **0.875^**^** | **-0.107** | **0.155** | **0.893^**^** | **0.812^**^** | **0.830^**^** | **0.792^**^** |
|  | **SR** | **1** | **0.945^**^** | **0.782^**^** | **0.782^**^** | **0.841^**^** | **0.754^**^** | **0.098** | **-0.323^*^** | **0.846^**^** | **0.811^**^** | **0.756^**^** | **0.689^**^** |
|  |  | **-** | **-0.022** | **-0.077** | **-0.059** | **0.005** | **-0.121** | **0.205** | **-0.478** | **-0.047** | **-0.001** | **-0.074** | **-0.103** |
| **RDW(g)** | **FR** | **0.967^**^** | **1** | **0.804^**^** | **0.791^**^** | **0.783^**^** | **0.845^**^** | **0.003** | **0.065** | **0.889^**^** | **0.776^**^** | **0.761^**^** | **0.718^**^** |
|  | **SR** | **0.945^**^** | **1** | **0.779^**^** | **0.779^**^** | **0.799^**^** | **0.706^**^** | **0.137** | **-0.348^*^** | **0.809^**^** | **0.764^**^** | **0.737^**^** | **0.684^**^** |
|  |  | **-0.022** | **-** | **-0.025** | **-0.012** | **0.016** | **-0.139** | **0.134** | **-0.413** | **-0.08** | **-0.012** | **-0.024** | **-0.034** |
| **SFW(g)** | **FR** | **0.859^**^** | **0.804^**^** | **1** | **0.979^**^** | **0.683^**^** | **0.702^**^** | **-0.142** | **0.180** | **0.703^**^** | **0.618^**^** | **0.767^**^** | **0.766^**^** |
|  | **SR** | **0.782^**^** | **0.779^**^** | **1** | **10.000^**^** | **0.722^**^** | **0.559^**^** | **0.110** | **-0.183** | **0.716^**^** | **0.686^**^** | **0.654^**^** | **0.591^**^** |
|  |  | **-0.077** | **-0.025** | **-** | **9.021** | **0.039** | **-0.143** | **0.252** | **-0.363** | **0.013** | **0.068** | **-0.113** | **-0.175** |
| **SDW(g)** | **FR** | **0.841^**^** | **0.791^**^** | **0.979^**^** | **1** | **0.657^**^** | **0.672^**^** | **-0.165** | **0.185** | **0.670^**^** | **0.601^**^** | **0.747^**^** | **0.742^**^** |
|  | **SR** | **0.782^**^** | **0.779^**^** | **10.000^**^** | **1** | **0.722^**^** | **0.559^**^** | **0.110** | **-0.183** | **0.716^**^** | **0.686^**^** | **0.654^**^** | **0.591^**^** |
|  |  | **-0.059** | **-0.012** | **9.021** | **-** | **0.065** | **-0.113** | **0.275** | **-0.368** | **0.046** | **0.085** | **-0.093** | **-0.151** |
| **RL (cm)** | **FR** | **0.836^**^** | **0.783^**^** | **0.683^**^** | **0.657^**^** | **1** | **0.987^**^** | **-0.408^**^** | **0.187** | **0.942^**^** | **0.971^**^** | **0.964^**^** | **0.930^**^** |
|  | **SR** | **0.841^**^** | **0.799^**^** | **0.722^**^** | **0.722^**^** | **1** | **0.834^**^** | **0.032** | **-0.246** | **0.952^**^** | **0.970^**^** | **0.913^**^** | **0.842^**^** |
|  |  | **0.005** | **0.016** | **0.039** | **0.065** | **-** | **-0.153** | **0.44** | **-0.433** | **0.01** | **-0.001** | **-0.051** | **-0.088** |
| **RSA (cm^2^)** | **FR** | **0.875^**^** | **0.845^**^** | **0.702^**^** | **0.672^**^** | **0.987^**^** | **1** | **-0.277^*^** | **0.146** | **0.984^**^** | **0.967^**^** | **0.936^**^** | **0.897^**^** |
|  | **SR** | **0.754^**^** | **0.706^**^** | **0.559^**^** | **0.559^**^** | **0.834^**^** | **1** | **-0.012** | **-0.333^*^** | **0.813^**^** | **0.786^**^** | **0.753^**^** | **0.689^**^** |
|  |  | **-0.121** | **-0.139** | **-0.143** | **-0.113** | **-0.153** | **-** | **0.265** | **-0.479** | **-0.171** | **-0.181** | **-0.183** | **-0.208** |
| **RAD (mm)** | **FR** | **-0.107** | **0.003** | **-0.142** | **-0.165** | **-0.408^**^** | **-0.277^*^** | **1** | **-0.344^*^** | **-0.122** | **-0.377^**^** | **-0.465^**^** | **-0.468^**^** |
|  | **SR** | **0.098** | **0.137** | **0.110** | **0.110** | **0.032** | **-0.012** | **1** | **-0.550^**^** | **0.314^*^** | **-0.022** | **0.047** | **0.059** |
|  |  | **0.205** | **0.134** | **0.252** | **0.275** | **0.44** | **0.265** | **-** | **-0.206** | **0.436** | **0.355** | **0.512** | **0.527** |
| **RLPV (cm/m^3^)** | **FR** | **0.155** | **0.065** | **0.180** | **0.185** | **0.187** | **0.146** | **-0.344^*^** | **1** | **0.092** | **0.193** | **0.231** | **0.263** |
|  | **SR** | **-0.323^*^** | **-0.348^*^** | **-0.183** | **-0.183** | **-0.246** | **-0.333^*^** | **-0.550^**^** | **1** | **-0.400^**^** | **-0.195** | **-0.164** | **-0.152** |
|  |  | **-0.478** | **-0.413** | **-0.363** | **-0.368** | **-0.433** | **-0.479** | **-0.206** | **-** | **-0.492** | **-0.388** | **-0.395** | **-0.415** |
| **RV (cm^3^)** | **FR** | **0.893^**^** | **0.889^**^** | **0.703^**^** | **0.670^**^** | **0.942^**^** | **0.984^**^** | **-0.122** | **0.092** | **1** | **0.932^**^** | **0.876^**^** | **0.833^**^** |
|  | **SR** | **0.846^**^** | **0.809^**^** | **0.716^**^** | **0.716^**^** | **0.952^**^** | **0.813^**^** | **0.314^*^** | **-0.400^**^** | **1** | **0.901^**^** | **0.871^**^** | **0.801^**^** |
|  |  | **-0.047** | **-0.08** | **0.013** | **0.046** | **0.01** | **-0.171** | **0.436** | **-0.492** | **-** | **-0.031** | **-0.005** | **-0.032** |
| **Tips** | **FR** | **0.812^**^** | **0.776^**^** | **0.618^**^** | **0.601^**^** | **0.971^**^** | **0.967^**^** | **-0.377^**^** | **0.193** | **0.932^**^** | **1** | **0.904^**^** | **0.888^**^** |
|  | **SR** | **0.811^**^** | **0.764^**^** | **0.686^**^** | **0.686^**^** | **0.970^**^** | **0.786^**^** | **-0.022** | **-0.195** | **0.901^**^** | **1** | **0.866^**^** | **0.821^**^** |
|  |  | **-0.001** | **-0.012** | **0.068** | **0.085** | **-0.001** | **-0.181** | **0.355** | **-0.388** | **-0.031** | **-** | **-0.038** | **-0.067** |
| **Forks** | **FR** | **0.830^**^** | **0.761^**^** | **0.767^**^** | **0.747^**^** | **0.964^**^** | **0.936^**^** | **-0.465^**^** | **0.231** | **0.876^**^** | **0.904^**^** | **1** | **0.976^**^** |
|  | **SR** | **0.756^**^** | **0.737^**^** | **0.654^**^** | **0.654^**^** | **0.913^**^** | **0.753^**^** | **0.047** | **-0.164** | **0.871^**^** | **0.866^**^** | **1** | **0.965^**^** |
|  |  | **-0.074** | **-0.024** | **-0.113** | **-0.093** | **-0.051** | **-0.183** | **0.512** | **-0.395** | **-0.005** | **-0.038** | **-** | **-0.011** |
| **Crossings** | **FR** | **0.792^**^** | **0.718^**^** | **0.766^**^** | **0.742^**^** | **0.930^**^** | **0.897^**^** | **-0.468^**^** | **0.263** | **0.833^**^** | **0.888^**^** | **0.976^**^** | **1** |
|  | **SR** | **0.689^**^** | **0.684^**^** | **0.591^**^** | **0.591^**^** | **0.842^**^** | **0.689^**^** | **0.059** | **-0.152** | **0.801^**^** | **0.821^**^** | **0.965^**^** | **1** |
|  |  | **-0.103** | **-0.034** | **-0.175** | **-0.151** | **-0.088** | **-0.208** | **0.527** | **-0.415** | **-0.032** | **-0.067** | **-0.011** | **-** |
